# Silencing of the Alkaline α-Galactosidase Gene *CsAGA1* Impairs Root and Gall Development in Cucumber upon *Meloidogyne incognita* Infection

**DOI:** 10.3390/ijms26146686

**Published:** 2025-07-11

**Authors:** Tingting Ji, Xingyi Wang, Xueyun Wang, Lihong Gao, Yongqiang Tian, Si Ma

**Affiliations:** 1 Beijing Key Laboratory of Growth and Development Regulation for Protected Vegetable Crops, College of Horticulture, China Agriculture University, Beijing 100193, China; jitingting@cau.edu.cn (T.J.); wangxyy@cau.edu.cn (X.W.); wxy15832324269@163.com (X.W.); gaolh@cau.edu.cn (L.G.); 2 Fujian Vegetable Engineering Technology Research Center, Fujian Key Laboratory of Vegetable Genetics and Breeding, Crop Research Institute, Fujian Academy of Agricultural Sciences, Fuzhou 350013, China

**Keywords:** *Cucumis sativus*, alkaline α-galactosidase, *Meloidogyne incognita*, sugar

## Abstract

*Meloidogyne incognita* (*M. incognita*) is a devastating root-knot nematode that parasitizes a broad range of crop species by inducing the formation of giant cells (GCs) in host roots, thereby facilitating nutrient acquisition. This process profoundly alters host sugar metabolism, yet the molecular regulators underlying sugar dynamics during infection remain poorly understood in cucumber. In this study, we investigated the role of the cucumber alkaline α-galactosidase gene (*CsAGA1*) in *M. incognita*-infected roots. Histochemical analysis of *proCsAGA1*::GUS transgenic lines demonstrated that *CsAGA1* is spatially localized to nematode-induced feeding sites, with its expression markedly induced in GCs and phloem-adjacent tissues during infection. Functional analyses revealed that silencing *CsAGA1* impaired root and gall development. *CsAGA1*-silenced plants exhibited increased gall numbers (per gram root) but significantly reduced root growth and smaller galls compared to controls. These results indicate that *CsAGA1* is required for proper gall expansion and root growth during *M. incognita* infection. This study provides novel insight into the sugar-mediated regulation of host–nematode interactions, and *CsAGA1* emerges as a potential target for the biological control of *M. incognita*.

## 1. Introduction

Cucumber (*Cucumis sativus* L.) is an important horticultural crop, valued for its high content of dietary fiber and minerals, and it is cultivated extensively worldwide. However, cucumber production is severely threatened by root-knot nematodes (RKNs, *Meloidogyne* spp.), which can substantially reduce both yield and quality. RKNs are obligate parasitic nematodes that infect plant roots, inducing the formation of specialized feeding structures that disrupt root development and impair nutrient and water uptake [1,2]. The primary RKN species include *Meloidogyne incognita* (*M. incognita*), *Meloidogyne javanica* (*M. javanica*), *Meloidogyne arenaria* (*M. arenaria*), and *Meloidogyne hapla* (*M. hapla*), among which *M. incognita* is considered the most destructive [3,4]. Despite the significant threat posed by RKNs, resistant cucumber cultivars and commercially available rootstocks for related cucurbit crops remain largely unavailable, underscoring the urgent need for the development of effective nematode management strategies.

The life cycle of root-knot nematodes can be completed within 20–40 days. The infective second-stage juveniles (J2s) invade the epidermal tissues of the root tip and subsequently migrate through the epidermal and cortical cells to reach suitable cells where they induce the formation of permanent feeding structures known as giant cells (GCs) [5]. GCs serve as the primary nutrient source for nematodes and exhibit high metabolic activity. Nematodes use their stylets to extract water, sugars, amino acids, and other essential nutrients from the GCs to support their growth, development, and reproduction [6,7]. Metabolomic analyses of *M. incognita*-infected alfalfa have revealed significant increases in starch, sucrose, glucose, malic acid, fumaric acid, and a range of amino acids, including valine, phenylalanine, aspartic acid, and glutamic acid, within the galls [7]. Sugars play a pivotal role in the interaction between nematodes and their host plants, serving as essential metabolites that facilitate both energy production and signaling pathways crucial for nematode development and pathogenicity [8]. Moreover, the modulation of sugar concentrations within the plant host can significantly influence the development of nematodes, thereby affecting the dynamics of plant–nematode interactions and potentially altering the outcomes of plant defense mechanisms [9]. In *Arabidopsis*, nematode infection leads to alterations in sugar metabolism pathways, suggesting that nematodes can manipulate the metabolic responses of the host to enhance their own survival [10]. Moreover, the role of sugars in plant innate immunity has been examined, revealing that sugars can modulate the defense responses of plants against nematode infections. The induction of resistance mechanisms in plants may be influenced by the availability and distribution of sugars, which can affect the plant’s ability to respond to nematode attack [11]. This interplay suggests that managing sugar levels could be a potential strategy for enhancing plant resistance to nematodes. Our previous study demonstrated the soluble sugar dynamics in nematode-infected cucumber. In infected cucumber roots, stachyose was significantly induced at the early stage of nematode infection, while stachyose and raffinose were dramatically decreased at the late stage of nematode infection [12].

α-Galactosidase (α-Gal) is a member of the glycoside hydrolase family and functions as an exoglycosidase, catalyzing the hydrolysis of α-galactosidic bonds with high efficiency. It primarily hydrolyzes raffinose family oligosaccharides (RFOs) into sucrose and galactose [13]. In cucumber, eight α-Gal genes have been identified, comprising four acidic and four basic isoforms [14]. Further characterization revealed that the basic isoforms exhibit a higher affinity for stachyose than raffinose, whereas the acidic isoforms show the opposite substrate preference [15]. Subcellular localization studies indicated that acidic α-galactosidase 1 (CsGal1) and CsGal2 are localized near the cell wall, while CsGal3 is localized within vacuoles. In contrast, the basic α-galactosidases (CsAGAs) are predominantly localized in the cytoplasm [15]. Functional studies have demonstrated that α-Gal enzymes play critical roles in plant growth and development, seed germination, sugar transport and unloading, and responses to abiotic stress [16]. In cucumber, *CsAGA1* expression progressively increases during fruit development, particularly within the vascular tissues of the fruit. Overexpression of *CsAGA1* results in a larger fruit size compared to the wild type, while disruption of *CsAGA1* and *CsAGA2* leads to delayed fruit development and significantly reduced levels of sucrose, glucose, and fructose [17]. *CsAGA2* is specifically involved in regulating the unloading of photosynthates from the phloem into the sieve element in cucumber fruits and in maintaining source–sink balance [18]. In *Arabidopsis*, overexpression of *ZmAGA1* from maize decreases RFOs and galactinol levels in mature seeds, promoting seed germination but compromising seed longevity [19]. In New Zealand spinach, the transcript levels of *TtAGA1* are significantly upregulated under abiotic stresses, including drought, salinity, and mechanical injury [20]. Furthermore, heterologous expression of *Vv-α-gal/SIP* in *Arabidopsis* has been shown to enhance salt tolerance, with transgenic plants exhibiting significantly improved germination and growth compared to wild-type plants [21]. Despite these advances, the role of AGAs/aGAs in plant responses to RKN infection has not yet been explored.

Cucumber is a typical RFO-transporting vegetable crop that transports RFOs as the primary form of translocated sugars [22]. RFOs have been shown to play crucial roles in plant growth, development, and responses to both abiotic and biotic stresses [23,24,25]. In our previous study, we investigated the transcriptional dynamics of sugar metabolism-related genes during *M. incognita* infection in cucumber [12]. Notably, the expression of *CsAGA1* was highly expressed in roots and was significantly upregulated by *M. incognita* infection. Histological analyses revealed that *CsAGA1* is specifically expressed in phloem tissues and GCs. Functional characterization through gene silencing showed that knockdown of *CsAGA1* led to a significant reduction in both the root growth and gall size. These findings suggest that *CsAGA1* may be involved in the *M. incognita* infection process by regulating gall development in cucumber. Overall, this study advances our understanding of the genetic mechanisms underlying *M. incognita* infection and identifies *CsAGA1* as a potential target for the development of novel biological control strategies against RKNs.

## 2. Results

### 2.1. CsAGA1 Is Significantly Upregulated in GCs with M. incognita Infection

**Figure 1:**
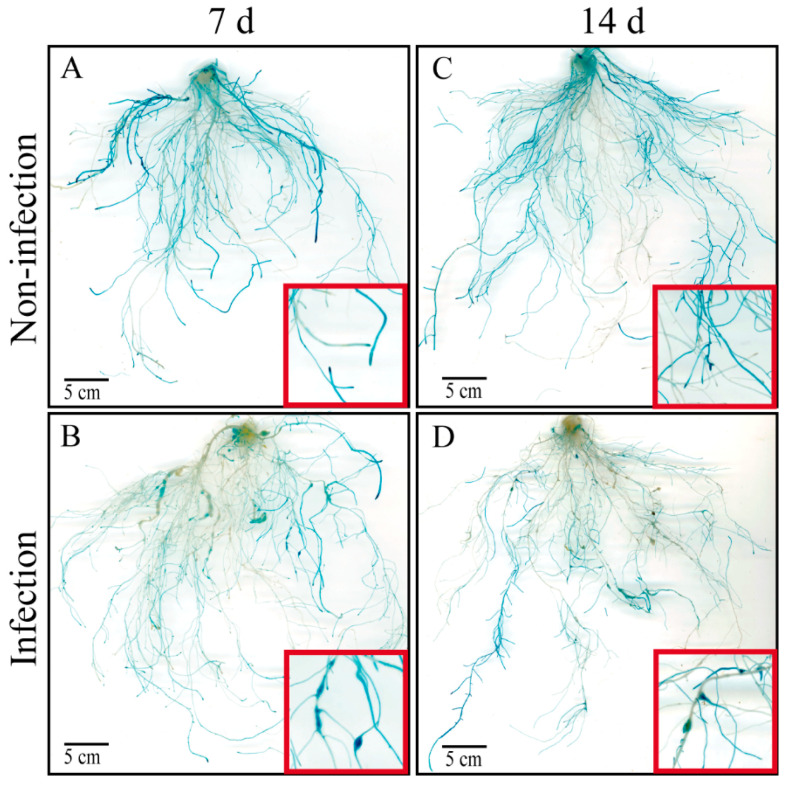
GUS staining patterns in hairy roots harboring *proCsAGA1::GUS* constructs, with or without *M. incognita* infection, detected at 7 dpi and 14 dpi. GUS localization of *CsAGA1* in hairy roots, without *M. incognita* infection, detected at 7 d (**A**) and 14 d (**C**). GUS localization of *CsAGA1* in hairy roots, with *M. incognita* infection, detected at 7 dpi (**B**) and 14 dpi (**D**). Scale bar = 5 cm. Red boxes indicate regions magnified view of roots.

**Figure 2:**
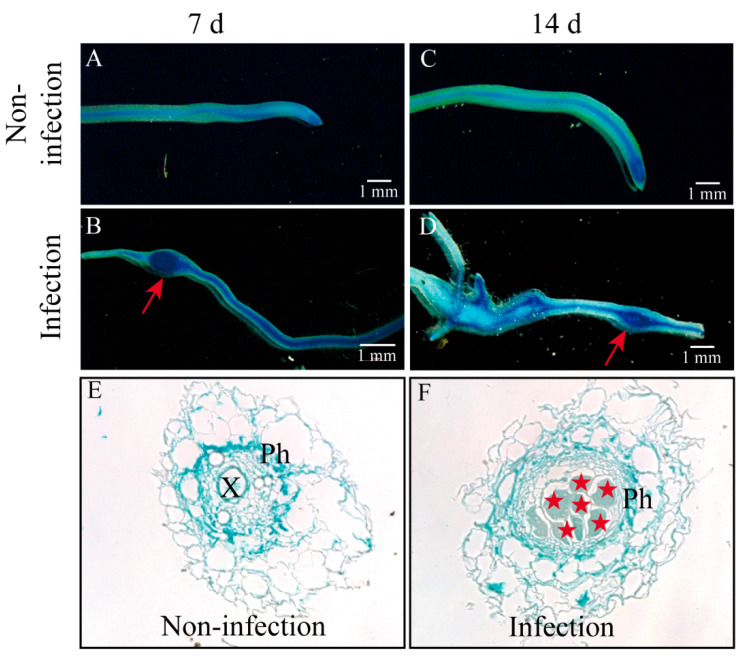
Expression of *proCsAGA1::GUS* in response to *M. incognita* infection in cucumber roots. GUS expression is present in roots, without *M. incognita* infection, detected at 7 d (**A**) and 14 d (**C**). Galls (indicated by red arrows) of *proCsAGA1::GUS* are observed in hairy roots, expressing at 7 dpi (**B**) and 14 dpi (**D**). GUS expression in 10 μm paraffin-embedded sections of roots without (**E**) or with (**F**) *M. incognita* infection for *proCsAGA1::GUS*. Annotations: red arrows indicate galls; pentagrams indicate GCs. Ph, phloem; X, xylem.

Our previous study found that the expression level of *CsAGA1* was significantly upregulated at 7, 14, 28, and 35 days post infestation (dpi) with *M. incognita*. To further investigate the tissue-specific localization of *CsAGA1* in cucumber roots during root-knot nematode infection, we generated *proCsAGA1::GUS* transgenic cucumber hairy roots and performed GUS staining following *M. incognita* infection at 7 and 14 dpi (Figure 1). Strong GUS staining driven by *proCsAGA1::GUS* was observed in cucumber roots (including root tips) and galls at 7 and 14 d (Figure 1 and Figure 2A–D). Paraffin sectioning displayed strong blue GUS signals in the GCs and surrounding phloem tissues (Figure 2E,F), indicating that CsAGA1 may play a role in giant cell development during nematode infection.

### 2.2. Down-Regulation of CsAGA1 Impairs Root Development in Cucumber

**Figure 3:**
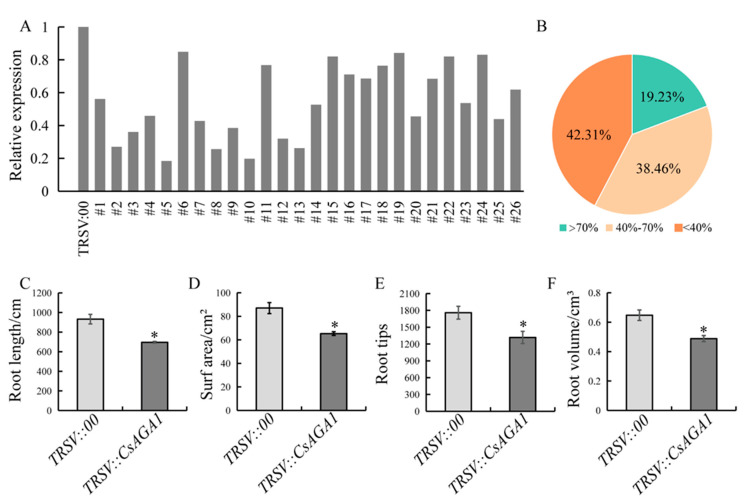
Silencing of *CsAGA1* via VIGS impairs root development in cucumber (**A**,**B**) Distribution of *CsAGA1* expression levels in individual VIGS-treated roots compared to the *TRSV::00* control. “>70%” indicates that *CsAGA1* expression was reduced to <30% of control levels (silencing efficiency >70%); “40–70%” corresponds to expression levels between 30 and 60% of the control; “<40%” indicates expression above 60% of control levels. (**C**–**F**) Quantitative analysis of root traits in *TRSV::00* and *TRSV::CsAGA1* plants at 14 dpi with *M. incognita*: root length (**C**), surface area (**D**), root tip number (**E**), and root volume (**F**). Data represent means ± SDs (*n* = 18). Significant differences are indicated as follows: * *p* < 0.05 (Student’s *t*-test).

**Figure 4:**
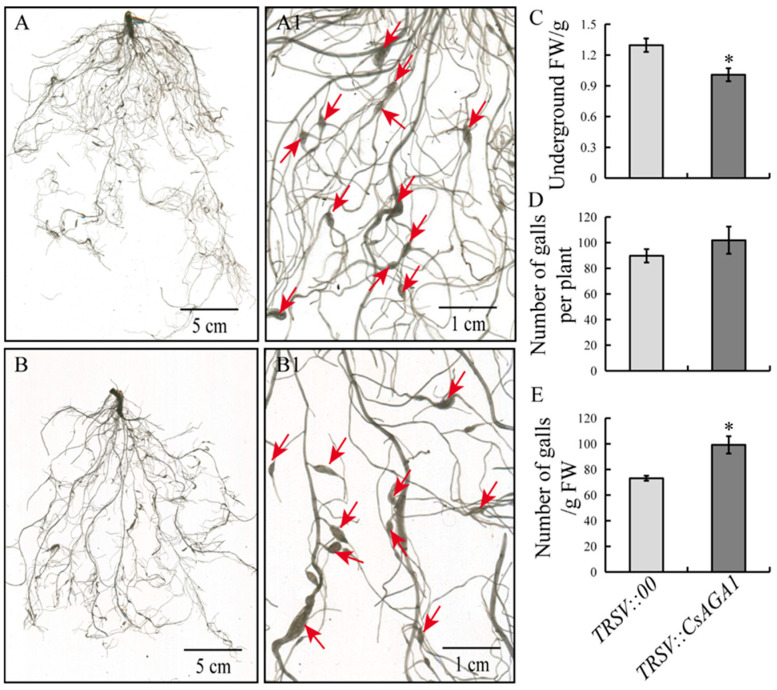
Suppression of *CsAGA1* through VIGS may not affect the infection by *M. incognita*. (**A**,**A1**) Root phenotype of control plants (*TRSV::00*) inoculated with *M. incognita*. (**B**,**B1**) Root phenotype of *CsAGA1*-silenced plants (*TRSV::CsAGA1*) inoculated with *M. incognita*. Comparison of underground weight (**C**), gall numbers per plant (**D**), and gall numbers/g FW root (**E**) in *TRSV::00* and *TRSV::CsAGA1* inoculated with nematodes at 14 dpi. Significant differences are indicated as follows: * *p* < 0.05 (Student’s *t*-test). Data are shown as mean ± SD (*n* = 18). Red arrows indicates the galls.

To investigate the functional role of *CsAGA1* in the root growth of cucumber, a *TRSV::CsAGA1* vector was generated, and *CsAGA1*-silenced plant lines were established. Silencing efficiency analysis revealed three distinct groups: 19.23% of lines showed ≥70% silencing efficiency, 38.46% exhibited moderate silencing (40–70%), and 42.31% demonstrated <40% efficiency (Figure 3A,B). Lines with silencing efficiency exceeding 40% (total 57.69%) were selected for subsequent phenotyping. *CsAGA1*-silenced plants exhibited statistically significant reductions across all measured parameters: total root length decreased by 25.3%, root surface area diminished by 25.0% (87.1 cm^2^ vs. 65.3 cm^2^), root volume declined by 24.5%, and underground fresh weight decreased by 22.2%. Additionally, root tip density showed a marked reduction of 25.1%, indicating compromised lateral root formation (Figure 3C–F and Figure 4C). The suppression of root development parameters strongly supports the essential role of *CsAGA1* in maintaining normal root system architecture.

### 2.3. CsAGA1 Silencing Impairs GCs Development

**Figure 5:**
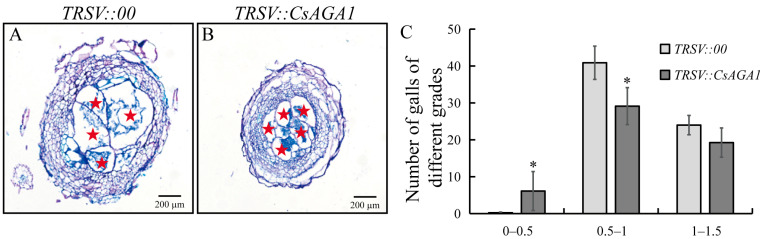
Suppression of *CsAGA1* through VIGS reduced gall sizes at 14 dpi with *M. incognita* infection. The sections of *TRSV::00* (**A**) and *TRSV::CsAGA1* (**B**) galls stained with toluidine blue. Pentagram indicates giant cell. Comparison of gall size abundance (**C**) in *TRSV::00* and *TRSV::CsAGA1* inoculated with nematode at 14 dpi. Significant differences are indicated as follows: * *p* < 0.05 (Student’s *t*-test). Data are shown as mean ± SD (*n* = 18).

To determine the role of *CsAGA1* in cucumber with *M. incognita* infection, we inoculated *M. incognita* in *TRSV::CsAGA1*-silenced plants and *TRSV::00* control plants. While gall numbers per plant showed no significant difference between *TRSV::CsAGA1* and *TRSV::00* control plants, gall numbers per gram root fresh weight in *TRSV::CsAGA1* were significantly increased compared to that in controls (Figure 4). Quantitative classification of nematode-induced galls at 14 dpi demonstrated a size distribution shift in silenced plants. *TRSV::CsAGA1* plants exhibited a 53.4% predominance of smaller galls (0.5–1.0 mm diameter), compared to 62.8% in controls. Conversely, control plants showed higher proportions of larger galls (>1.0 mm diameter; 36.9% vs. 35.3% in silenced plants) (Figure 5C). Cellular-level examination revealed compromised GC development in silenced plants. The average GC diameter decreased in *TRSV::CsAGA1*, correlating with reduced gall dimensions (Figure 5A,B). These findings suggest that *CsAGA1* silencing disrupts normal GC expansion rather than initial nematode penetration. The impaired GC development is likely to create suboptimal feeding sites, potentially explaining the observed developmental delay in gall size.

## 3. Discussion

RKNs are obligate plant parasites that pose a serious threat to global agricultural productivity and cause substantial economic losses [26]. During the early stages of infection, J2s invade the plant root system, typically through the root tip, and induce the differentiation of vascular parenchyma cells into multinucleated GCs at their feeding sites [5]. These GCs serve as specialized nutrient sinks, providing sugars, proteins, and other essential metabolites to support nematode growth and development [6,7,27]. In tomato, for example, sucrose delivered from the phloem accumulates in developing galls as glucose and fructose levels rise over time [28]. Similarly, we observed, in cucumber roots, an early spike in RFOs followed by their late-stage decline, while sucrose fell initially and glucose surged later [12]. These dynamic sugar patterns imply an initial mobilization of RFOs followed by conversion into simple sugars to fuel the feeding site. The consistent upregulation of *CsAGA1* throughout infection (7–35 dpi) suggests that it drives this RFO-to-glucose shift at the feeding site [12].

In this study, *CsAGA1* expression was detected in GCs and the nearby phloem, consistent with a role in local RFO hydrolysis. By analogy to known RFO translocation mechanisms, *CsAGA1* likely splits phloem-delivered RFOs into sucrose and galactose at the feeding site [29]. Functional disruption of *CsAGA1* strongly impaired feeding site development (Figure 5). Silencing *CsAGA1* reduced gall and GC sizes, indicating that its activity is needed to establish a robust nutrient sink. This phenotype parallels reports in other cucurbits: for example, knockout of a watermelon AGA2 gene blocks RFO hydrolysis and diminishes sugar accumulation in fruit [30]. Likewise, interference with RFO metabolism enzymes often causes growth defects in plants [29]. In the galls induced by the nematode, limiting RFO catabolism would restrict the supply of hexoses needed by both the plant cells and the parasite. With less sucrose cleavage by SUS and fewer hexoses generated, GCs cannot expand fully, and nematode development is expected to stall [28]. Thus, *CsAGA1* appears to help convert stored RFOs into the sugars that feed GC growth and nematode nutrition.

In higher plants, α-galactosidases (α-Gals) participate in many developmental and stress response processes, but their roles in root development remain largely uncharacterized [16,18,19,24,31]. In this study, cucumber plants silenced for *CsAGA1* showed dramatically inhibited root growth under nematode stress, with significantly reduced root length, fresh weight, and volume (Figure 3 and Figure 4). This phenotype points to a critical role for *CsAGA1* in supporting root development. Indeed, sugar availability is known to govern root architecture: for example, sucrose and glucose promote lateral root initiation by modulating auxin biosynthesis and signaling pathways [32,33]. Likewise, stress-induced enhancement of sucrose flux to roots can stimulate root growth under drought; *Arabidopsis* SWEET11/12 transporters are phosphorylated, which increases sucrose import into roots, raises root sucrose content, and in turn improves root elongation and branching [34]. *CsAGA1* silencing may limit the pool of mobile sugars, thus restricting sucrose supply to the root. Insufficient sucrose in roots could blunt auxin-related gene expression or transport and thereby suppress auxin-driven lateral root formation.

Remarkably, CsAGA1-silenced plants did not differ from controls in nematode gall number per plant (Figure 4D). This mirrors the *Arabidopsis atsuc2* mutant phenotype: *atsuc2* plants form normal numbers of galls but show arrested nematode development due to impaired sucrose transport [35]. Thus, *CsAGA1* appears to affect gall maturation and nutrition rather than the initial infection process. We propose that *CsAGA1* is required to maintain the sucrose–auxin balance in stressed roots, ensuring adequate sugar provision for auxin-mediated root development even during nematode challenge.

## 4. Materials and Methods

### 4.1. Plant Materials and Growth Conditions

A cucumber inbred line (*Cucumis sativus* L. ‘Xintaimici’) was used in this study. Cucumbers with *proCsAGA1::GUS* transgenic hairy roots were grown in a controlled growth chamber at 25 °C with a 16 h light/8 h dark photoperiod. *CsAGA1*-silenced cucumber plants were cultivated in an incubator programmed to 22 °C during the 16 h light period and 18 °C during the 8 h dark period. Nematode egg masses were collected from galls of water spinach (*Ipomoea aquatica*), incubated in water at 28 °C for a week, and the resulting pre-parasitic second-stage juveniles (pre-J2s) were collected and used for cucumber root inoculation.

### 4.2. RNA Expression Analysis

Root and gall samples were collected from cucumber with or without *M. incognita* infection at 7 dpi and 14 dpi. Root surfaces were thoroughly rinsed with DEPC-treated water to remove residual soil and sand. For each time point, six biological replicates were collected. Samples (0.1 g) were flash-frozen in liquid nitrogen and ground thoroughly, and total RNA was extracted using the Plant RNA Extraction Kit (Promega, Shanghai, China). Real-time quantitative PCR (qPCR) was performed using the ChamQ SYBR qPCR Master Mix (LowROX Premixed) kit (Novozymes, Nanjing, China). *CsUBI* (Csa2G036600) was used as an internal reference, and each sample was analyzed with three biological replicates. Gene expression levels were calculated using the 2^−ΔΔCT^ method for relative quantification.

### 4.3. GUS Localization Analysis

The promoter region of *CsAGA1* was cloned and inserted into the *PstI* and *BamHI* restriction sites of the pCAMBIA1319 vector and subsequently transformed into the *Agrobacterium rhizogenes* strain K599 (Weidi Biotechnology, Shanghai, China). Transgenic hairy roots carrying the *proCsAGA1::GUS* construct were generated following the protocol described by Zhang et al. (2023) [36]. Each *proCsAGA1::GUS* hairy root was inoculated with 300 pre-J2s of *M. incognita*. Root samples were collected at 7 and 14 dpi, along with uninoculated controls, for GUS staining. Samples were incubated in GUS staining solution at 37 °C for 90 min and subsequently decolorized with 70% ethanol. GUS staining patterns were documented using both a camera and a stereo microscope. For detailed observation, gall and control samples were embedded in paraffin, sectioned using a microtome, and imaged under an Olympus CX23 microscope (Yijingtong Optical Technology Co., Ltd., Guangzhou, China). The primers are listed in Supplementary Table S1.

### 4.4. VIGS Assay and Phenotypic Observation

A ~300 bp specific fragment from the coding sequence of *CsAGA1* was amplified and inserted into the *TRSV2* vector, as previously described by Fang et al., 2021 [37]. The *Agrobacterium tumefaciens* strains used for virus-induced gene silencing (VIGS) were cultured and activated. Cucumber seeds were surface-sterilized and germinated on Murashige and Skoog (MS) medium. After two days, the seeds were immersed in an infection solution composed of an equal-volume mixture of *TRSV1* and *TRSV2::CsAGA1* bacterial suspensions, followed by vacuum infiltration for 20 min. The treated seeds were then transferred to sterile filter paper to remove excess liquid and subsequently placed on solid medium containing acetosyringone. The seeds were incubated in the dark for 2–3 days until visible bacterial growth was observed around the seeds. The infected seedlings were then transplanted into a 1:1 (*v*/*v*) sand-to-vermiculite mixture. Once the control (*TRSV::CsPDS*) plants exhibited an albino phenotype, nematode inoculation was carried out using 200 pre-parasitic second-stage juveniles (pre-J2s) per plant. Phenotypic observations and physiological measurements were conducted 14 dpi. Primers are provided in Supplementary Table S1.

### 4.5. Structure Observation of Paraffin

The collected samples were fixed in FAA fixative solution (50% FAA) under a vacuum for 20 min, then transferred to fresh fixative and incubated for 24 h. Samples were dehydrated using a graded ethanol series on a shaker, followed by clearing with xylene, and subsequently embedded in paraffin wax. Sections were cut using a paraffin microtome and stained with 50% toluidine blue solution. Stained sections were visualized and imaged using an Olympus microscope.

### 4.6. Statistical Analysis

Experimental data were processed using Microsoft Excel 2016 for basic statistical calculations, and significance analysis was performed using SPSS 21.0. A Student’s *t*-test was employed to evaluate statistical differences between groups. Data are presented as the means  ±  standard deviations (SDs) from independent biological replicates.

## 5. Conclusions

In summary, our data identify *CsAGA1* as a potential mediator of sugar remodeling in cucumber galls induced by *M. incognita*. Silencing *CsAGA1* impaired root and gall development, implying that CsAGA1-driven RFO catabolism is crucial for establishing the sugar-rich environment required by the nematode. Modulating this pathway may therefore offer a strategy to weaken nutrient supply for nematode development. Future work should explore how CsAGA1 interacts with sucrose transporters (SWEETs, SUTs) and invertases in galls, and whether breeding or biotechnological control of RFO metabolism can enhance resistance to RKNs.

## Data Availability

All data are presented in the main manuscript and Supplementary Materials.

## Supplementary Materials

The following supporting information can be downloaded at: https://www.mdpi.com/article/10.3390/ijms26146686/s1.
