# Supplementary material for: Silencing of the Alkaline α-Galactosidase Gene CsAGA1 Impairs Root and Gall Development in Cucumber upon Meloidogyne incognita Infection
Source: Int J Mol Sci. 2025 Jul 11;26(14):6686. doi: 10.3390/ijms26146686 (PMC12294682; doi:10.3390/ijms26146686)
Supplement: Supplementary file 1 [file ijms-26-06686-s001.zip › ijms-3706808-supplementary.pdf]

Supporting information

Supplemental Table S1. Primers used in this study.

Gene	ID	Primer	Sequence
CsUBI	Csa2G036600	Q-CsUBI-F	CCTTATTGACCAACCAGTAGT
		Q-CsUBI-R	GGACAATGTTGATTTCCTCG
AGA1	Csa4G631570	Q-CsAGA1-F	TGGTGGTGTTTGTTTTAGCG
		Q-CsAGA1-R	CCAGAGCATCAGCGGTTT
		CsAGA1-VIGS-F	TGTTTTAAATGCCTTTACGTACGACCGACAAAGGACTGCC
		CsAGA1-VIGS-R	AACACACAAAACACCTACGTACCCCCGTGATCGTGTCG
		CsAGA1-1391-F	gcgcgccaagcttggctgcagAATGTTATGACGAAGCAAACGAGA
		CsAGA1-1391-R	tcttagaattcccggggatccAGGCGTGCAATGGTAGTTGG
